# Genome-Wide and Candidate Gene Association Study of Cigarette Smoking Behaviors

**DOI:** 10.1371/journal.pone.0004653

**Published:** 2009-02-27

**Authors:** Neil Caporaso, Fangyi Gu, Nilanjan Chatterjee, Jin Sheng-Chih, Kai Yu, Meredith Yeager, Constance Chen, Kevin Jacobs, William Wheeler, Maria Teresa Landi, Regina G. Ziegler, David J. Hunter, Stephen Chanock, Susan Hankinson, Peter Kraft, Andrew W. Bergen

**Affiliations:** 1 Division of Cancer Epidemiology and Genetics, NCI, Bethesda, Maryland, United States of America; 2 Program in Molecular and Genetic Epidemiology, Harvard School of Public Health, Boston, Massachusetts, United States of America; 3 Johns Hopkins Bloomberg School of Public Health, Department of Biostatistics, Baltimore, Maryland, United States of America; 4 Channing Laboratory, Brigham and Womens' Hospital, Boston, Massachusetts, United States of America; 5 Core Genotyping Facility, Advanced Technology Center, National Cancer Institute, Gaithersburg, Maryland, United States of America; 6 BioInformed LLC, Gaithersburg, Maryland, United States of America; 7 Information Management Services, Inc., Rockville, Maryland, United States of America; 8 Molecular Genetics Program, Center for Health Sciences, SRI International, Menlo Park, California, United States of America; Leiden University Medical Center, Netherlands

## Abstract

The contribution of common genetic variation to one or more established smoking behaviors was investigated in a joint analysis of two genome wide association studies (GWAS) performed as part of the Cancer Genetic Markers of Susceptibility (CGEMS) project in 2,329 men from the Prostate, Lung, Colon and Ovarian (PLCO) Trial, and 2,282 women from the Nurses' Health Study (NHS). We analyzed seven measures of smoking behavior, four continuous (cigarettes per day [CPD], age at initiation of smoking, duration of smoking, and pack years), and three binary (ever versus never smoking, ≤10 versus >10 cigarettes per day [CPDBI], and current versus former smoking). Association testing for each single nucleotide polymorphism (SNP) was conducted by study and adjusted for age, cohabitation/marital status, education, site, and principal components of population substructure. None of the SNPs achieved genome-wide significance (p<10^−7^) in any combined analysis pooling evidence for association across the two studies; we observed between two and seven SNPs with p<10^−5^ for each of the seven measures. In the chr15q25.1 region spanning the nicotinic receptors *CHRNA3* and *CHRNA5*, we identified multiple SNPs associated with CPD (p<10^−3^), including rs1051730, which has been associated with nicotine dependence, smoking intensity and lung cancer risk. In parallel, we selected 11,199 SNPs drawn from 359 *a priori* candidate genes and performed individual-gene and gene-group analyses. After adjusting for multiple tests conducted within each gene, we identified between two and five genes associated with each measure of smoking behavior. Besides *CHRNA3* and *CHRNA5*, *MAOA* was associated with CPDBI (gene-level p<5.4×10^−5^), our analysis provides independent replication of the association between the chr15q25.1 region and smoking intensity and data for multiple other loci associated with smoking behavior that merit further follow-up.

## Introduction

Cigarette smoking is a risk factor for more than two dozen diseases and the single biggest cause of preventable mortality worldwide [1]. Although public awareness of the dangers of smoking is widespread and public health measures such as public building smoking restrictions and increased cigarette taxes have had salutary effects on smoking rates, dependence on nicotine, the major psychoactive component in tobacco, induces most people who start smoking to continue to smoke in spite of their wish to quit [2]. Environmental influences on tobacco dependence including cultural perceptions and economics, low socioeconomic status, peer smoking and maternal smoking during pregnancy are well documented. Nevertheless, twin studies provide strong evidence that a range of diverse smoking phenotypes including age at initiation, intensity, and cessation have a substantial hereditary component 1, 3, 4, 5, 6. Identifying the specific loci that influence smoking behaviors (including initiation, intensity and cessation) could lead to important etiological insights and facilitate the development of treatments to further reduce smoking related mortality.

Genome-wide linkage studies have identified chromosomal regions that may harbor loci contributing to one of many smoking behavior phenotypes: age at initiation [7], [8]; some variant of CPD [9], [10], [11], [12], [13], [14], [15], [16], [17] DSM-IV Nicotine Dependence [11], [17]; some variant of Ever-Never [9], [17], [18], [19], [20]; Fagerstrom test for nicotine dependence (FTQ, FTND) or Heaviness Smoking Index (HIS) [14], [21], [22]; Pack-years [9], [23]; Current versus Former [11]; and withdrawal severity [11]. While some regions have shown suggestive linkage to smoking behaviors in multiple studies [24], linkage results have generally been heterogeneous and short on conclusive findings; to date no risk loci have been discovered that definitively account for linkage signals.

Until very recently, candidate gene association studies have focused on genes in a few candidate pathways. A ‘reward deficiency syndrome’ has been postulated as one unifying theme to account for the role of diverse neurotransmitters in nicotine dependency [25], [26], [27], and consequently many studies have evaluated genes in opioid [28], [29], serotinergic [30], [31], [32], dopaminergic [26], [33], [34], [35], drug metabolizing enzyme [36], [37], [38], [39], [40], [41] and nicotinic and muscarinic cholinergic receptor pathways[42], [43]. Results from these studies have been largely equivocal, due to small sample sizes in individual studies, incomplete and non-overlapping genetic coverage, differences in measures of smoking behavior, or differences in genetic and environmental backgrounds. It is also highly probable that many of the loci that influence smoking behavior lie outside of the previously-studied candidate regions.

A recent genome-wide association study of over 13,000 smokers identified a region on chromosome 15q25.1 associated with smoking intensity (number of cigarettes smoked per day) [44]. This region, spanning the nicotinic acetylcholine receptors, *CHRNA5*, and *CHRNA3*, and *CHRNB4* and was also identified in a recent GWAS of dichotomized smoking intensity [45], and in two genome-wide association scans of lung cancer [46], [47], It was unclear whether the association between SNPs in this region and lung cancer was due to a genetic effect on smoking behavior, an independent effect on lung carcinogenesis, or both [48]. Two recent candidate gene studies together including almost 5000 smokers both found SNPs in nicotinic receptors including the chr15p25.1 nicotinic receptor loci to be associated with nicotine dependence [49], [50].

To identify loci associated with smoking initiation, intensity and cessation we performed a genome-wide association study (GWAS) using data from subjects genotyped as part of the Cancer Genetic Markers of Susceptibility (CGEMS) project, including 2,617 ever-smokers [51], [52]. In addition to single-marker tests of association in the GWAS, we also report results from gene- and gene-group-level tests of association of 359 candidate genes in 30 functional groups.

## Methods

### Samples and genotypes

Subjects were drawn from two previous genome-wide association studies (GWAS), performed as part of the Cancer Genetic Markers of Susceptibility (CGEMS) project [51], [52]. Data on smoking behaviors were available on 2,060 men from the Prostate, Lung, Colon and Ovarian Trial (PLCO) (1,172 prostate cancer cases and 1,157 controls) and on 2,282 postmenopausal women (1,145 with breast cancer and 1,142 controls) from the Nurses' Health Study (NHS). All subjects were of self-reported European ancestry, which was consistent with genetic analyses of population structure [53]. Samples from the PLCO were genotyped using the Illumina HumanHap 300 k and HumanHap 240 k platforms [52]; those from the NHS were genotyped using the Illumina HumanHap 550 k platform [51]. Genotyping was performed at the same laboratory and similar genotyping quality control (QC) procedures were used in each study. Individual samples were removed if more than 10% of SNPs failed genotyping, and individual SNPs were removed if more than 10% of samples failed. The average call rate for both PLCO and NHS samples was 99.8%. Combined genome-wide analyses were restricted to directly-genotyped SNPs with minor allele frequencies above 1% in each study (ca. 518,000 SNPs). Additional description of these studies is available in previous reports [51], [52].

### Adjustment for population stratification

For both PLCO and NHS, analyses of population stratification were conducted using approximately 10,000 unlinked SNP markers [51], [52]. Individual European, Asian and African admixture proportions were estimated by STRUCTURE [53] applied to CGEMS data augmented by the HapMap CEU, CHB+JPT and YRI samples. Subjects with significant non-European admixture were excluded for PLCO and NHS. Residual within-Europe population stratification was estimated using the top three (PLCO) or four (NHS) principal components of genetic variation, as calculated using EIGENSTRAT [54].

### Smoking behaviors

We selected four continuous and three binary smoking behaviors for analysis (Table 1). The continuous measures were cigarettes smoked per day (CPD), age at smoking initiation (SMKAGE), duration of smoking (SMKDU) and pack-years (PKYRS); the binary measures were ever versus never smoking status (EVNV), smokers who quit versus those who did not (CIGSTAT), and a binary measure of smoking intensity (CPDBI, defined as ten or more cigarettes per day versus fewer than ten). Only current or former smokers were included in the analyses involving the smoking phenotypes with the exception of EVNV which included never smokers.

**Table 1:** The distribution of smoking behaviors and covariates in NHS and PLCO CGEMS samples.

Smoking Behaviors or Covariates *	NHS (n = 2282)	PLCO (n = 2060)
CPD (Cigarettes per day, mean±SD)	18.5±10.5	22.0±13.6
SMKAGE (Age of initiation, years, mean±SD)	19.6±3.7	18.1±5.0
SMKDU (Duration, years, mean±SD)	25.3±15.5	27.7±13.8
PKYRS (Pack-years, mean±SD)	24.9±22.2	31.8±27.3
EVNV (N, %)		
*Ever smokers*	1244 (55)	1373 (67)
*Never smokers*	1038 (45)	687 (33)
CIGSTAT (N, %)		
*Former smokers*	1107 (89)	1161 (85)
*Current smokers*	137 (11)	212 (15)
CPDBI (N, %)		
>10 CPD	906 (75)	1167 (85)
*≤10 CPD*	305 (25)	202 (15)
Living Status (N, %)		
*Alone/not married*	457 (20)	271 (13)
*Not alone/married*	1825 (80)	1789 (87)
Education (N, %)		
*No college*	0	838 (41)
*Some college*	1591 (70)	409 (20)
*Bachelors*	463 (20)	392 (19)
*Masters or above*	228 (10)	421 (20)
Age (N, %)		
*55–59* years	111 (5)	400 (19)
*60–64*	437 (19)	648 (31)
*65–69*	411 (18)	651 (32)
*70–74*	581 (26)	361 (18)
*>74*	742 (32)	0

* descriptive statistics for smoking behaviors included ever smokers only.

All of these behaviors were measured by baseline questionnaire (BQ) in the PLCO (administered from 1994–2001) [55]. Age at initiation was defined as the age when a subject started smoking “regularly for six months or longer.” Former smokers were defined as ever-smokers who did not smoke regularly at BQ and were asked to report the age at which they stopped smoking regularly. Ever smokers were asked to provide information on the number of cigarettes they smoked per day, in categories (1–10, 11–20, 21–30, 31–40, 41–60, 61–80, over 80). For continuous analyses we assigned subjects to the midpoint of their category (or 90 cigarettes per day for over 80). Duration was derived from data on age at smoking initiation and age at cessation. Pack years was derived by converting cigarettes per day to packs per day (CPD/20) and multiplying this figure by duration.

In the NHS, SMKAGE was measured at BQ; all other behaviors were measured using cumulative information from the BQ (administered in 1976) and subsequent follow-up questionnaires (one every two years). The majority of NHS subjects (2149) had smoking data available up through the 2002 questionnaire. For those few women (133) with no smoking data available from the 2002 follow-up cycle, we used data from the latest available follow-up. Age at initiation was defined as the age when a subject started smoking cigarettes “regularly.” Former smokers were defined as ever-smokers who identified themselves as non-smokers on any questionnaire (and did not identify as a smoker on any subsequent questionnaire). Age at cessation was explicitly asked in NHS BQ. For women who quit smoking after the BQ, age at cessation was inferred as the median age between the questionnaire that defined the woman as a former smoker and the last questionnaire that identified her as a smoker. Prior to 1982, current or former smokers were asked to write in the average number of cigarettes they smoked per day; subsequent questionnaires captured information about smoking intensity in categories (1–4, 5–14, 15–24, 25–34, 35–44, and over 44 cigarettes per day). Pack years was estimated as the sum of the products of smoking intensity (categories were assigned midpoint values, e.g. 5–14 was coded as 10 cigarettes per day, or 0.5 packs per day) at questionnaire k times the interval between questionnaire k and questionnaire k+1 (or half that interval for women who were smokers at questionnaire k and non-smokers at questionnaire k+1). Smoking duration was calculated as the sum of all intervals where a woman was a smoker. Average cigarettes per day (the CPD variable used in this GWAS) was calculated as pack-years divided by smoking duration.

### Association analyses

Continuous phenotypes were log transformed to achieve approximate normality and SNP genotypes were coded as counts of minor alleles. For each study, we defined any phenotype that was more than three standard deviations from the mean to be an outlier. Outliers that were above (below) the mean were then truncated to the 99th (1st) percentile of the raw distribution. We tested for association between each SNP marker and each continuous phenotype using linear regression, adjusted for study center (PLCO) or geographic region (NHS); age at smoking assessment in five-year bins (baseline for PLCO or last available follow-up for NHS); marital status (married versus not; PLCO) or living arrangement (living alone or with others, NHS); education (4 categories PLCO, 3 categories NHS); prostate (PLCO) or breast (NHS) cancer case-control status; and selected principal components of genetic variation. For binary traits, we used unconditional logistic regression, adjusted for the same covariates. These tests were conducted separately for PLCO and NHS. For SNPs that passed QC filters and had minor allele frequency above 1% in both studies, we combined evidence for association across PLCO and NHS using weighted Z-scores [56]. Heterogeneity in SNP-smoking behavior associations across study was assessed using *Q* and *I* ^2^ statistics [57]. Power calculations were performed using Quanto (http://hydra.usc.edu/GxE/)[58]

### Analyses of candidate genes and candidate gene groups

We selected 359 genes for additional analyses, based on their hypothesized relationship to smoking behavior. 349 of these genes were previously selected by the NICSNP Candidate Gene Committee [50]. We added ten candidate genes identified from two recent GWAS of dichotomized measures of nicotine dependence (*CTNNA3*, *FBXL17*, *FTO*, *NRX1*, *PBX2*, *TRPC7*, *VPS13A*) and CPD (*DGK1*, *RORB*, *SLCO3A1*) [45], [59].

For each candidate gene we tested the null hypothesis that no SNP within 20 kb upstream of the start of transcription and 10 kb downstream of the stop of transcription (based on NCBI build 35.1) was associated with smoking behaviors using a parametric permutation procedure that allows for covariate adjustment. We compared the smallest observed p-value for any SNP in the candidate gene region to the empirical null distribution of the smallest p-value based on 20,000 random permutations. This approach provides a gene-level p-value that is adjusted for both the number of SNPs in the gene region and their linkage disequilibrium structure.

Candidate genes (and the SNPs in the corresponding gene regions) were grouped based on known functional similarity. We used a slightly modified version of the groups developed by the NICSNP Candidate Gene Committee. (Table S1). To test for association between SNPs in a group and smoking behaviors, we used a modified rank truncated product method [60] which compares the product of the ten smallest gene-level p-values over all the genes in the group to its simulated null distribution. Such a group or pathway level analysis potentially has more power to detect associations when a group containing multiple susceptibility genes each has modest evidence for association [61].

### Chr15q25.1 SNP imputation

Multiple SNPs in the 15q25.1 region have been shown to be associated with CPD, nicotine dependence, or risk of lung cancer [44], [47], [49], [50], [59]. We directly genotyped some of these SNPs and could impute others using the observed genotypes in PLCO and NHS samples and the phased HapMap CEU samples (Release 21). Imputation, restricted to the region of chromosome 15q23, was performed for each study separately using the Hidden Markov Model implemented in Mach 1.0 [62]. All of the imputed genotypes had high quality scores (R-squares>0.8 for 95% of SNPs in the region).

## Results

The distributions of the smoking behaviors and demographic covariates included in the analysis of the NHS and PLCO datasets are presented in Table 1. The men in the PLCO sample have smoking behaviors that are more prevalent and more severe (greater frequency of ever, current and heavy smokers, earlier age of onset, longer duration, and greater pack-years) than do the women in the NHS sample. The direction and significance of correlations among the smoking phenotypes within the dataset are similar, with all correlations highly significant (P<0.0001), except for the correlation between age at initiation and cigarettes per day in the NHS sample (Table S2).

**Figure 1:**
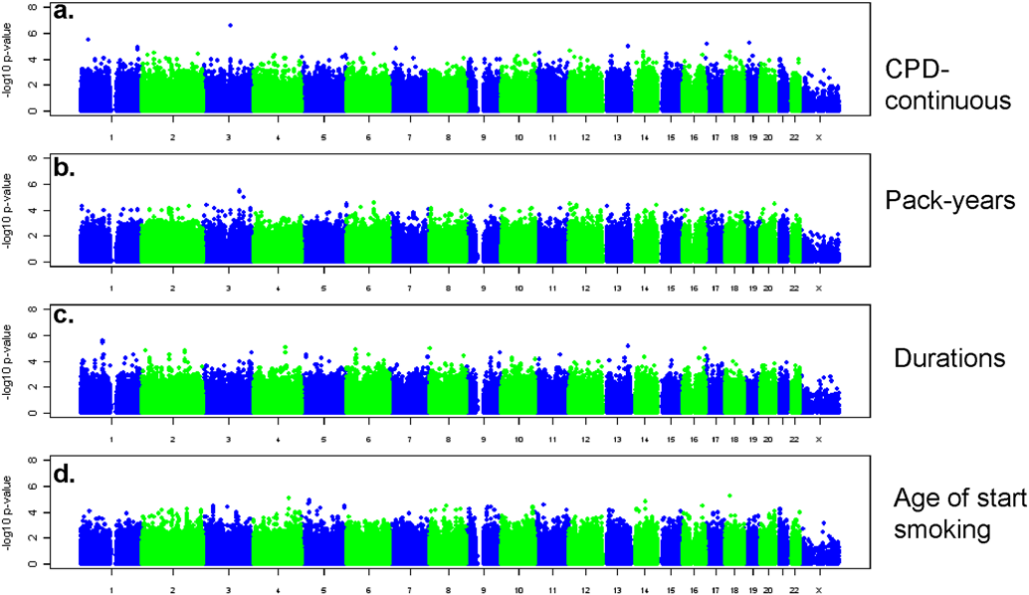
−log_10_ p-values for association with seven continuous (Figure 1) and categorical (Figure 2) smoking behaviors. P-values are based on the combined evidence for association from both PLCO and NHS.

**Figure 2:**
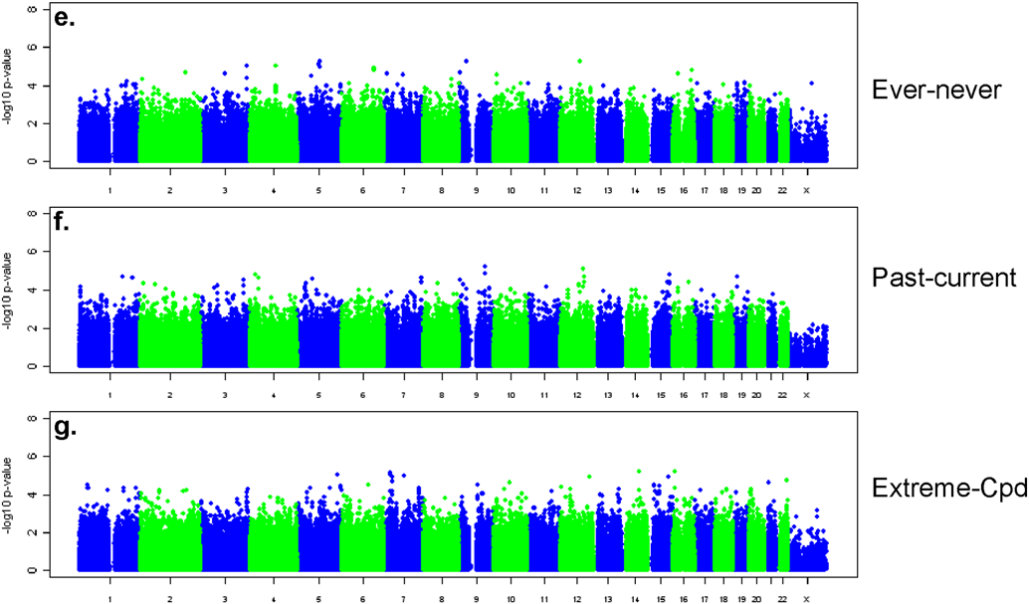
−log_10_ p-values for association with seven continuous (Figure 1) and categorical (Figure 2) smoking behaviors. P-values are based on the combined evidence for association from both PLCO and NHS.

Quantile-quantile plots of the −log_10_ p-values for SNP association with smoking behaviors (Figure S1) showed no evidence for systematic bias (each genomic inflation factor λ<1.02). None of the SNPs achieved genome-wide significance (p<10^−7^) in any combined analysis pooling evidence for association across the two studies (Figures 1 and 2). Table 2 lists detailed results for SNPs with a combined-analysis p-value<10^−5^ for each smoking behavior. For the combined GWAS analysis of the seven smoking behaviors, the most significant SNP smoking behavior association result is rs6437740 with CPD (P = 2.4×10^−7^). Including this result, there are 8 gene regions and 3 genomic regions with predicted but not verified coding regions associated with SNPs in the group of SNP smoking behavior results with P<10^−5^ (Table 2). We observed no evidence for systematic heterogeneity in results between studies, and no single SNP showed evidence for heterogeneity by sex at the genome-wide significance level (see summary of Q statistics in Figure S2).

**Table 2:** SNPs with P_weight_<10^−5^ for seven smoking behaviors in 4,611 men and women of European ancestry.

Smoking Behavior	SNP	Chr	Coordinate	Gene Region	maf NHS	maf PLCO	beta NHS	beta PLCO	P NHS	P PLCO	P weight	Q
CPD	rs6437740	3	108,948,507	BBX	0.23	0.24	−0.14	−0.10	3.70E-05	1.30E-03	2.40E-07	0.90
	rs910696	1	30,236,689		0.30	0.31	0.11	0.08	2.20E-04	3.30E-03	3.00E-06	0.64
	rs10411195	19	19,897,176	ZNF505	0.02	0.03	−0.37	−0.21	2.80E-04	5.10E-03	5.80E-06	1.52
	rs7050529	X	110,961,378	TRPC5	0.06	0.07	−0.28	−0.06	2.80E-06	7.30E-02	6.20E-06	9.75
	rs758642	17	3,733,656	CAMKK1	0.35	0.34	0.10	0.08	7.00E-04	3.00E-03	7.30E-06	0.21
SMKAGE	rs11082304	18	18,974,971	CABLES1	0.49	0.49	0.02	0.03	2.70E-03	6.80E-04	6.00E-06	0.70
	rs17050782	4	140,780,739	SET7	0.21	0.21	0.02	0.03	4.50E-03	5.60E-04	8.40E-06	1.17
SMKDU	rs7553864	1	87,325,379	AK002179	0.41	0.39	0.12	0.08	9.80E-04	8.40E-04	2.70E-06	0.52
	rs719015	1	87,323,731	AK002179	0.42	0.40	0.13	0.07	2.70E-04	3.60E-03	3.80E-06	1.56
	rs912969	13	102,665,105		0.07	0.07	−0.24	−0.14	8.50E-04	2.70E-03	7.80E-06	1.19
	rs950063	4	126,789,524		0.38	0.39	−0.12	−0.08	1.50E-03	1.90E-03	9.00E-06	0.68
PKYRS	rs800082	3	145,822,903		0.44	0.42	0.16	0.12	1.00E-03	1.00E-03	3.30E-06	0.48
	rs9289678	3	145,789,794		0.43	0.42	0.16	0.12	1.20E-03	9.60E-04	3.70E-06	0.42
EVNV	rs1402279	12	76,231,853		0.05	0.05	−0.52	−0.41	1.80E-04	7.30E-03	5.20E-06	0.27
	rs933688	5	90,798,504	LOC133789	0.15	0.17	0.20	0.39	1.60E-02	5.10E-05	5.70E-06	2.14
	rs1889899	9	26,779,940		0.39	0.37	0.20	0.23	1.60E-03	1.10E-03	5.70E-06	0.16
	rs6452953	5	90,758,790		0.16	0.17	0.20	0.39	1.80E-02	5.60E-05	7.00E-06	2.18
	rs6862125	5	90,760,354		0.15	0.17	0.20	0.39	2.00E-02	5.90E-05	8.50E-06	2.23
	rs6444087	3	187,104,531		0.26	0.26	0.22	0.25	1.40E-03	2.10E-03	9.20E-06	0.05
	rs1400363	4	104,608,326		0.42	0.42	−0.15	−0.26	1.30E-02	1.40E-04	9.90E-06	1.59
CIGSTAT	rs10989661	9	101,702,423		0.25	0.26	−0.30	−0.52	4.50E-02	1.50E-05	6.30E-06	1.37
	rs1847461	12	89,579,976		0.06	0.06	−0.84	−0.59	4.50E-04	4.90E-03	8.20E-06	0.64
	rs10859032	12	89,599,693		0.06	0.06	−0.84	−0.58	4.40E-04	5.10E-03	8.40E-06	0.67
CPDBI	rs3112740	16	7,776,298		0.14	0.14	0.38	0.77	1.00E-02	1.50E-04	6.00E-06	2.38
	rs2268983	14	68,478,450	ACTN1	0.48	0.49	−0.24	−0.42	1.10E-02	1.60E-04	6.70E-06	1.43
	rs3027409	X	43,363,287	MAOA	0.05	0.05	−0.50	−0.55	1.40E-02	1.10E-04	6.70E-06	0.04
	rs886716	7	26,330,858		0.32	0.31	−0.26	−0.42	9.60E-03	2.10E-04	7.70E-06	1.13
	rs4722613	7	26,385,573	LOC441205	0.13	0.14	−0.38	−0.50	5.00E-03	5.70E-04	9.30E-06	0.38

Maf = minor allele frequency.

Beta = per-minor-allele change in the mean trait value for continuous traits, and the per-minor-allele change in odds for binary traits.

p-weight = the meta-analysis p-value for the combined NHS and PLCO, using Stouffer's method.

Q = test for heterogeneity in genetic effect between studies.

We analyzed 359 candidate genes previously nominated as candidate genes for nicotine dependence [50], or identified in GWAS studies of dichotomized nicotine dependence and CPD [45], [59] and gene-level results are summarized in Table 3. Of note, rs3027409 in the *MAOA* candidate gene region had a p-value of 6.7×10^−6^ for association with CPDBI, which led to a gene-level p-value smaller than 5.4×10^−5^, the smallest *a priori* candidate gene association we observed. Nine candidate gene groups were associated with at least one smoking behavior at the 0.10 level (Table 4) with two (Nicotinic Receptors and Voltage-Dependent Calcium-Activated Potassium Channels) associated with a smoking behavior (CPD) at the 0.01 level.

**Table 3:** Candidate gene results with gene-level P<0.01 for seven smoking behaviors.

Smoking Behavior	Candidate Gene Region^1^	N SNPs	Significant SNP	GWAS P_weight_	Permuted P	beta PLCO	beta NHS
CPD	MAOA	8	rs2235186	3.87E-04	1.90E-03	0.03	0.11
	TRPV1	28	rs4790520	9.52E-05	2.43E-03	0.07	0.10
	CHRNA3	15	rs12914385	3.20E-04	3.05E-03	0.05	0.09
	CHRNA5	8	rs1051730	6.13E-04	3.10E-03	0.04	0.09
	FOSB	5	rs2238686	1.63E-03	6.86E-03	−0.09	−0.09
SMKAGE	SLC1A2	55	rs16927393	2.89E-05	1.10E-03	0.05	0.05
	CYP2A6	2	rs8102683	1.57E-03	2.76E-03	−0.03	−0.01
	RYR1	33	rs8107027	2.32E-04	7.14E-03	0.01	0.08
	CHRNA1	8	rs2646165	1.45E-03	9.29E-03	0.01	0.04
SMKDU	GRM1	49	rs12197749	9.81E-05	3.24E-03	0.07	0.15
	NCOA1	11	rs9309308	3.74E-04	3.48E-03	0.06	0.13
	ADCY3	27	rs2278483	4.46E-04	7.29E-03	0.06	0.12
PKYRS	MAOA	8	rs2235186	6.09E-05	1.90E-04	0.06	0.18
	GRM6	12	rs1845940	3.68E-05	3.33E-04	0.13	0.14
	PIK3C2G	84	rs2305220	8.04E-05	5.14E-03	0.10	0.15
	GPR51	158	rs1000440	4.67E-05	5.33E-03	0.07	0.19
	CHRNA3	15	rs12914385	1.04E-03	9.29E-03	0.09	0.11
EVNV	CYP2B6	13	rs2014141	7.31E-05	7.36E-04	0.11	0.25
	SLC6A3	23	rs464049	2.61E-04	5.79E-03	0.13	0.20
CIGSTAT	GRPR	2	rs12845178	1.20E-03	1.24E-03	0.12	0.48
	NR3C2	82	rs10050229	1.47E-04	8.69E-03	−0.32	−0.40
CPDBI	MAOA	8	rs3027409	6.73E-06	5.36E-05*	−0.55	−0.50
	ATM	9	rs609261	7.16E-04	4.81E-03	0.15	0.34
	FOSB	5	rs2238686	2.22E-03	8.29E-03	−0.42	−0.20

* Bonferroni P value, as 20,000 permutations were too few to accurately determine the p-value in that range.

1 Gene regions can overlap, so some SNPs may be in multiple gene regions.

**Table 4:** Candidate Gene Groups with gene-level P<0.10.

Phenotype	Candidate gene group	N Genes	P value
CPD	Nicotinic Receptors	16	0.005
CPD	Voltage-Dependent Calcium-Activated Potassium Channels	7	0.008
SMKAGE	Cytochrome P450s	5	0.032
SMKAGE	Neuropeptides	10	0.062
SMKDU	Cytochrome P450s	5	0.049
SMKDU	Nicotinic Receptors	16	0.061
SMKDU	Opioid and Opioid-Like Neuropeptides	8	0.072
EVNV	Cytochrome P450s	5	0.027
EVNV	Alcohol Dehydrogenases	7	0.045
CIGSTAT	Cell Cycle Control	6	0.039
CPDBI	Muscarinic Receptors	3	0.062
CPDBI	Dopamine	12	0.074

**Figure 3:**
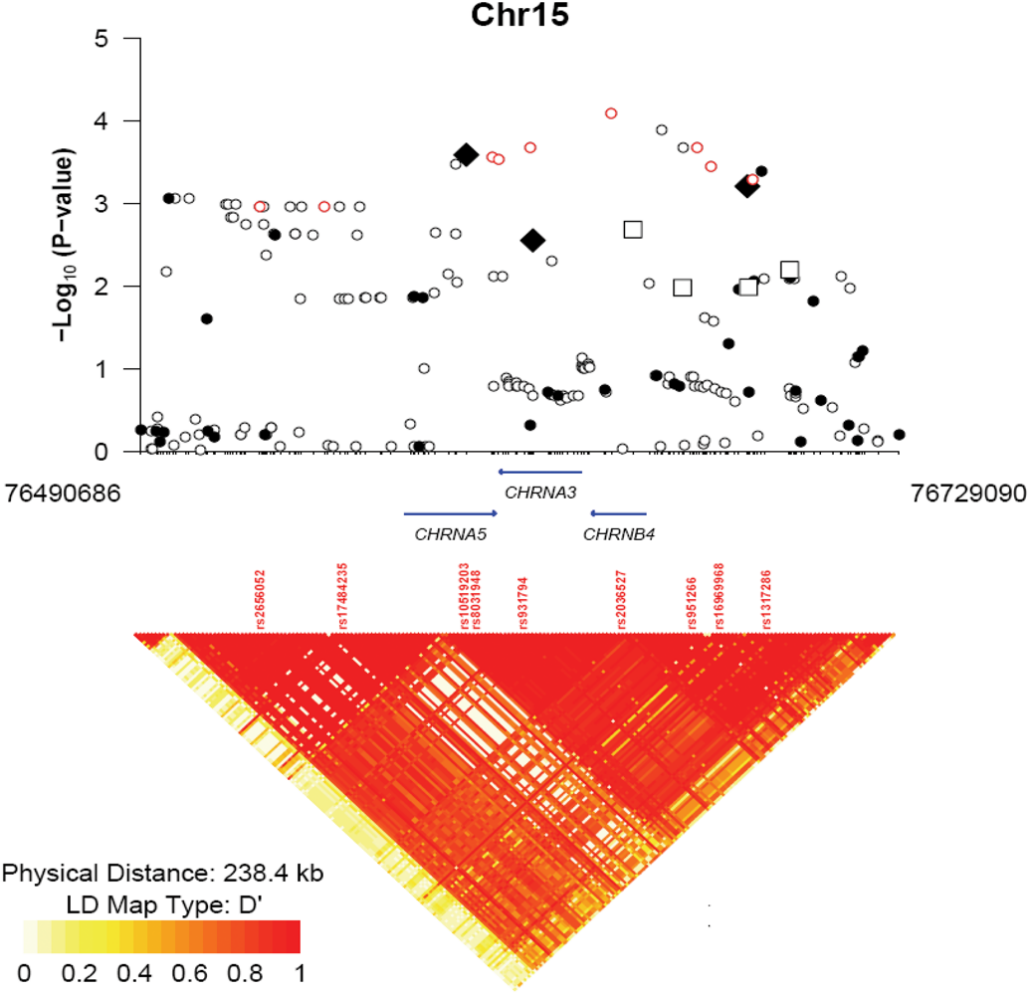
−log_10_ p-values for association with number of cigarettes smoked per day (CPD) for SNPs in the chr15q25.1 region. P-values are based on the combined evidence for association from both PLCO and NHS. Filled symbols denote genotyped SNPs; open symbols denote imputed SNPs. Black diamonds (squares) denote SNPs associated with continuous (binary) CPD in previous reports. Red circles denote SNPs associated with lung cancer in previous reports.

Figure 3 and Table S3 summarize the associations between genotyped and imputed SNPs and CPD in PLCO and NHS smokers for the chr15q25.1 region spanning *CHRNA3* and *CHRNA5*. The strongest association signal we observe in this region is at rs2036527 (combined P = 8×10^−5^), located 10,051 base pairs 3′ of *PSMA4* and 6,290 base pairs 5′ of *CHRNA5* in a region of strong linkage diseqilibrium spanning both genes.

## Discussion

We performed genome wide association analyses for seven related smoking behaviors in two datasets totaling 4,611 individuals and 2617 ever smokers. We selected smoking behaviors with established hereditary components [4], [5], [21], [63] and public health relevance[64], [65]. To the best of our knowledge this study represents the first genome-wide association study of duration of smoking, pack years, and age at initiation of smoking. The sample size is also larger than most published candidate gene association studies of smoking behavior [66] and two previous genome-wide association studies of smoking behaviors [45], [59].

Although we did not discover novel genome-wide significant (p<10^−7^) associations, we did find additional evidence for an association between genetic variants in the chr15q25.1 region and number of cigarettes smoked per day. Candidate gene analyses also provided suggestive evidence for association between variants in the *MAOA* gene region and the smoking behavior cigarettes per day.

The lack of genome-wide significant results suggests that common variants have at most a modest influence on smoking behavior. We had adequate power to detect a variant that explained even 2.5% of the variation in cigarettes per day. We had 61% power in the NHS sample and 71% power in the PLCO sample to detect such a variant at the 10^−7^ level; the power of the combined analysis was greater than 99%. Conversely, the lack of genome-wide significant findings does not rule out the existence of (many) common variants with small individual effects on smoking behavior, since our power to detect any one is small. Even with our relatively large sample size, our power to detect a variant similar to the 15q25.1 SNP rs1051730 (which was estimated to explain about 0.7% of the trait variance [44] at the genome-wide significance level) was only 8.5% for the combined analysis (and less than 1% for either study alone).

SNPs at the nicotinic receptor candidate genes *CHRNA3* (chr15q25.1) and *CHRNA1* (chr2q31.1) are associated in the CGEMS sample with three smoking behaviors: CPD, PKYRS and SMKAGE (Table 3). Another candidate gene association study investigating 348 of 359 candidate genes included in this study [50] evaluated association with a dichotomized nicotine dependence phenotype, and identified nicotinic receptor SNPs associated with FTND, including rs578776 and rs1051730 within *CHRNA3*, and rs16969968 within *CHRNA5*. Nicotinic receptors are also associated with CPD in the candidate gene group analysis as the most significantly associated gene group, and also with the phenotype SMKDU (Table 4).

Finally, we combined our chr15q25.1 results with data from three other published reports (Table S3) [44], [46], [47]. The SNP rs1051730, found within *CHRNA3* (Ex5+268), was highly statistically significantly associated with CPD (p = 5×10^−32^); the SNP rs8034191 (*LOC123688* IVS2+256) was also highly statistically significantly associated with CPD (p = 2×10^−29^). These SNPs were evaluated using a total of 26,789 (rs1051730) or 24,891 (rs8034191) smokers from this study and two other reports. The *CHRNA5* SNP rs16969968 (Ex5-54, D398N) was significantly associated (p<.01) with CPD in this study but not an earlier, smaller study; combined evidence for association in 3,464 smokers remained significant (p = 2×10^−3^). Comparative judgments of the relative importance of the individual SNPs are not possible due to the different sample sizes, the strong LD among the SNPs and the inability to adjust for the effects of the other SNPs in this meta-analysis.

Our candidate gene analyses identified an association (rs3027409, p<5.4×10^−5^) between genetic variation in *MAOA* and a dichotomized measure of smoking intensity (10 or less cigarettes smoked per day versus more than 10). This was the only gene-level result that remained significant after Bonferroni correction for the number of genes tested, which we regard as a conservative multiple-testing correction. This association is notable because of the role of the monoamine oxidases in the regulation of catecholamines and the inhibition of monoamine oxidases A and B by tobacco smoke [67]. There is substantial evidence that smoking results in reduced levels of the monoamine oxidase enzymes [67], [68] and subsequent reduced catabolism of dopamine likely contributes to the reinforcing and motivating effects of smoking. Investigation of MAO-related polymorphisms in relation to alcoholism [69], [70], Parkinson disease [71], [72], [73] and smoking [34], [67], [70], [74], [75], [76], [77] have yielded mixed results; our results suggest further investigation of this X-chromosome locus is warranted.

The gene group analysis that we performed provides one way to summarize the statistical evidence for association between a trait and multiple genetic variants across groups of genes that share sequence similarity and function. Nicotinic cholinergic receptors and voltage-dependent calcium-activated potassium channels were significantly associated with CPD (gene group P<0.01). We have previously discussed nicotinic receptor findings above. The association of rs7050529 (IVS3+286 of TRPC5) with CPD (Table 2) is notable as a closely related family member, *TRPC7*, was previously significantly associated with nicotine dependence [59]. The transient receptor potential cation family is a superfamily of 28 genes coding for cationic ion channels responding to temperature, endogenous and exogenous organic compounds, Ca2+ flux, and mechanical stimuli, and are expressed in nearly every tissue [78]. This study, the NICSNP study and Feng et al., 2006 [79] have identified significant associations between five Transient Receptor Family Potential (TRP) subfamily members and nicotine related behaviors in the canonical (this study Table 2, [59] Table 1, and [79]) and vanilloid subfamilies (this study Table 3, and Saccone et al., 2007, Supplementary Material [50]). Recently, Gu et al., 2005 [80] have shown that vanilloid subfamily members are expressed in the lung and are responsible for the pulmonary chemoreflex response, suggesting further study of these TRP subfamilies and their potential role in smoking behavior and downstream consequences may be fruitful.

The cytochrome P450, cell cycle control, and alcohol dehydrogenase candidate genes groups also exhibited nominally significant (0.01<P_permuted_<0.05) associations with smoking behaviors (Table 4). The cytochrome P450 results may have been driven by association between SNPs at *CYP2B6* with EVNV, and *CYP2A6* and SMKAGE (Table 3). These results are consistent with evidence for the relationship between *CYP2A6* genetic variation and both nicotine metabolism [81], [82], [83] and smoking behavior [41], [84].

In our study, the observed association between cell cycle control genes and quit status may be driven by association of SNPs at *FBXL17* (gene-level, p = 0.021, rs1433050) and *NFKB1* (rs10489113, gene-level P = 0.022). *FBXL17* is one of 68 members of the human F-box protein superfamily, a large group of ubiquitin ligases [85]. Ubiquitin ligases function in the ubiquitin-proteasome complex, which regulates protein assembly, trafficking and degradation, a cellular activity itself regulated by nicotine [86]. *FBXL17* was also identified in the NICSNP GWAS [59] as significantly associated with FNTD, via another SNP (rs10793832). None of the SNPs in the same high linkage-disequilibrium bin as rs10793832 (according to the Pelegen genome browser) were in high linkage disequilibrium with rs1433050, the *FBXL17* SNP identified in this study. One SNP genotyped in this study (rs885624) was in the same LD block as rs10793832 but was not significantly associated with quit status in either this study alone or in the combined analysis (p = 0.39).

The finding that the alcohol dehydrogenases genes were significantly associated with the smoking behavior EVNV in this analysis (e.g., *ADH4* gene-level P = 0.048 (rs3828541), and *ADH6*, gene-level P = 0.034 (rs3857224) suggests that genetic variation at these ADH loci may influence the establishment of smoking behavior. However this analysis did not control for alcohol consumption and so this finding should be considered preliminary.

Because of the large number of male and female smokers, we were able to conduct genome-wide association scans stratified by gender (study), and conduct a genome-wide association scan for differences in genetic effect between men and women. Such analyses are important, because the effect for some loci may differ between men and women or be restricted to one gender, e.g., due to differences in the environment. However, no SNPs achieved genome-wide significance for association with any smoking behavior in either study, and no SNP achieved genome-wide significance for heterogeneity in effect between men and women (between studies).

This study has several strengths. We performed a GWAS and candidate gene study investigating a variety of smoking behaviors with public health importance for the first time in a sample unselected for smoking behaviors and/or smoking attributable disease. We confirm important findings from recent GWAS and candidate gene studies of nicotine dependence and CPD. Our sample size is relatively large, yet still not large enough to reliably detect variants with modest effects on smoking behaviors. The absence of selection bias in the cohort bases for the samples enhances generalizability to U.S. non-Hispanic whites although a modest limitation is that the education level in both cohorts is above average. By limiting analyses to subjects of European ancestry and adjusting for principal components of population structure, we minimized risk of false positives due to population stratification, but are not be able to detect SNP alleles associated with smoking behavior that are common in non-Europeans but rare among European-Americans. The smoking behavior characteristics for the two studies are quite similar after taking into account expected differences by gender (Table 1), and the correlation of smoking behaviors are similar within NHS and PLCO (see Table S1). The combined sample has the advantage of increased power and generalizablity.

The diverse smoking behaviors we investigated represent the spectrum of key events in an individual's smoking history from initiation (age at initiation, ever never smoking) thru establishment of dependency (smoking duration, smoking intensity, and pack years), to outcome (current versus former cigarette smoking status), with potential genetic influence at each stage. The finding that selected genes are associated with multiple phenotypes may represent both correlations among the phenotypes but also pleiotropic effects of the genes, and is a strength of the integrative approach [87]. Although we did not identify specific candidate regions that achieved the genomewide threshold of statistical significance, our study provides candidate genes for follow-up evaluation. Future GWAS studies with additional smoking behavioral measures, including nicotine dependence measures, the planned sharing of data across large consortia with increased sample size [88] and the functional analysis of individual SNPs [89], will be required to achieve the necessary power and specificity to understand SNP with low effects (OR<1.3), effects in subgroups, explore effect modification by demographic variables, and dissect pleiotropy.

## Supporting Information

Table S1 (0.04 MB XLS)

Table S2 (0.03 MB DOC)

Table S3 (0.05 MB DOC)

Figure S1 (0.43 MB TIF)

Figure S2 (0.31 MB TIF)

## References

[1] Bergen AW, Caporaso N (1999). Cigarette smoking.. J Natl Cancer Inst.

[2] Ronald M, Davis TEN, Willaim RLynn (1988). The Health Consequences of Smoking: Nicotine Addiction: A Report of the Surgeon General: Center for Health Promotion and Education, Office on Smoking and Health, United States Public Health Service, Office of the Surgeon General.

[3] Batra V, Patkar AA, Berrettini WH, Weinstein SP, Leone FT (2003). The genetic determinants of smoking.. Chest.

[4] Li MD (2006). The genetics of nicotine dependence.. Curr Psychiatry Rep.

[5] Maes HH, Sullivan PF, Bulik CM, Neale MC, Prescott CA (2004). A twin study of genetic and environmental influences on tobacco initiation, regular tobacco use and nicotine dependence.. Psychol Med.

[6] Lessov CN, Martin NG, Statham DJ, Todorov AA, Slutske WS (2004). Defining nicotine dependence for genetic research: evidence from Australian twins.. Psychol Med.

[7] Vink JM, Beem AL, Posthuma D, Neale MC, Willemsen G (2004). Linkage analysis of smoking initiation and quantity in Dutch sibling pairs.. Pharmacogenomics J.

[8] Vink JM, Posthuma D, Neale MC, Eline Slagboom P, Boomsma DI (2006). Genome-wide linkage scan to identify Loci for age at first cigarette in Dutch sibling pairs.. Behav Genet.

[9] Bergen AW, Korczak JF, Weissbecker KA, Goldstein AM (1999). A genome-wide search for loci contributing to smoking and alcoholism.. Genet Epidemiol.

[10] Lessov-Schlaggar CN, Pergadia ML, Khroyan TV, Swan GE (2008). Genetics of nicotine dependence and pharmacotherapy.. Biochem Pharmacol.

[11] Swan GE, Hops H, Wilhelmsen KC, Lessov-Schlaggar CN, Cheng LS (2006). A genome-wide screen for nicotine dependence susceptibility loci.. Am J Med Genet B Neuropsychiatr Genet.

[12] Li MD, Ma JZ, Payne TJ, Lou XY, Zhang D (2008). Genome-wide linkage scan for nicotine dependence in European Americans and its converging results with African Americans in the Mid-South Tobacco Family sample.. Mol Psychiatry.

[13] Li MD, Payne TJ, Ma JZ, Lou XY, Zhang D (2006). A genomewide search finds major susceptibility loci for nicotine dependence on chromosome 10 in African Americans.. Am J Hum Genet.

[14] Sullivan PF, Neale BM, van den Oord E, Miles MF, Neale MC (2004). Candidate genes for nicotine dependence via linkage, epistasis, and bioinformatics.. Am J Med Genet B Neuropsychiatr Genet.

[15] Moslehi R, Goldstein AM, Beerman M, Goldin L, Bergen AW (2003). A genome-wide linkage scan for body mass index on Framingham Heart Study families.. BMC Genet.

[16] Saccone SF, Pergadia ML, Loukola A, Broms U, Montgomery GW (2007). Genetic linkage to chromosome 22q12 for a heavy-smoking quantitative trait in two independent samples.. Am J Hum Genet.

[17] Gelernter J, Liu X, Hesselbrock V, Page GP, Goddard A (2004). Results of a genomewide linkage scan: support for chromosomes 9 and 11 loci increasing risk for cigarette smoking.. Am J Med Genet B Neuropsychiatr Genet.

[18] Bierut LJ, Rice JP, Goate A, Hinrichs AL, Saccone NL (2004). A genomic scan for habitual smoking in families of alcoholics: common and specific genetic factors in substance dependence.. Am J Med Genet A.

[19] Ehlers CL, Wilhelmsen KC (2006). Genomic screen for loci associated with tobacco usage in Mission Indians.. BMC Med Genet.

[20] Pomerleau OF, Pomerleau CS, Chu J, Kardia SL (2007). Genome-wide linkage analysis for smoking-related regions, with replication in two ethnically diverse populations.. Nicotine Tob Res.

[21] Li MD (2003). The genetics of smoking related behavior: a brief review.. Am J Med Sci.

[22] Li MD, Sun D, Lou XY, Beuten J, Payne TJ (2007). Linkage and association studies in African- and Caucasian-American populations demonstrate that SHC3 is a novel susceptibility locus for nicotine dependence.. Mol Psychiatry.

[23] Faraone SV, Su J, Taylor L, Wilcox M, Van Eerdewegh P (2004). A novel permutation testing method implicates sixteen nicotinic acetylcholine receptor genes as risk factors for smoking in schizophrenia families.. Hum Hered.

[24] Li MD (2008). Identifying susceptibility loci for nicotine dependence: 2008 update based on recent genome-wide linkage analyses.. Hum Genet.

[25] Blum K, Braverman ER, Holder JM, Lubar JF, Monastra VJ (2000). Reward deficiency syndrome: a biogenetic model for the diagnosis and treatment of impulsive, addictive, and compulsive behaviors.. J Psychoactive Drugs.

[26] Blum K, Sheridan PJ, Wood RC, Braverman ER, Chen TJ (1996). The D2 dopamine receptor gene as a determinant of reward deficiency syndrome.. J R Soc Med.

[27] Comings DE, Blum K (2000). Reward deficiency syndrome: genetic aspects of behavioral disorders.. Prog Brain Res.

[28] Lerman C, Wileyto EP, Patterson F, Rukstalis M, Audrain-McGovern J (2004). The functional mu opioid receptor (OPRM1) Asn40Asp variant predicts short-term response to nicotine replacement therapy in a clinical trial.. Pharmacogenomics J.

[29] Ray R, Jepson C, Wileyto EP, Dahl JP, Patterson F (2007). Genetic variation in mu-opioid-receptor-interacting proteins and smoking cessation in a nicotine replacement therapy trial.. Nicotine Tob Res.

[30] Lerman C, Caporaso NE, Audrain J, Main D, Boyd NR (2000). Interacting effects of the serotonin transporter gene and neuroticism in smoking practices and nicotine dependence.. Mol Psychiatry.

[31] Lerman C, Shields PG, Audrain J, Main D, Cobb B (1998). The role of the serotonin transporter gene in cigarette smoking.. Cancer Epidemiol Biomarkers Prev.

[32] O'Gara C, Knight J, Stapleton J, Luty J, Neale B (2008). Association of the serotonin transporter gene, neuroticism and smoking behaviours.. J Hum Genet.

[33] Blum K, Sheridan PJ, Wood RC, Braverman ER, Chen TJ (1995). Dopamine D2 receptor gene variants: association and linkage studies in impulsive-addictive-compulsive behaviour.. Pharmacogenetics.

[34] McKinney EF, Walton RT, Yudkin P, Fuller A, Haldar NA (2000). Association between polymorphisms in dopamine metabolic enzymes and tobacco consumption in smokers.. Pharmacogenetics.

[35] Shields PG, Lerman C, Audrain J, Bowman ED, Main D (1998). Dopamine D4 receptors and the risk of cigarette smoking in African-Americans and Caucasians.. Cancer Epidemiol Biomarkers Prev.

[36] Benowitz NL, Swan GE, Jacob P, Lessov-Schlaggar CN, Tyndale RF (2006). CYP2A6 genotype and the metabolism and disposition kinetics of nicotine.. Clin Pharmacol Ther.

[37] Caporaso NE, Lerman C, Audrain J, Boyd NR, Main D (2001). Nicotine metabolism and CYP2D6 phenotype in smokers.. Cancer Epidemiol Biomarkers Prev.

[38] Fujieda M, Yamazaki H, Saito T, Kiyotani K, Gyamfi MA (2004). Evaluation of CYP2A6 genetic polymorphisms as determinants of smoking behavior and tobacco-related lung cancer risk in male Japanese smokers.. Carcinogenesis.

[39] Kamataki T, Fujieda M, Kiyotani K, Iwano S, Kunitoh H (2005). Genetic polymorphism of CYP2A6 as one of the potential determinants of tobacco-related cancer risk.. Biochem Biophys Res Commun.

[40] Malaiyandi V, Sellers EM, Tyndale RF (2005). Implications of CYP2A6 genetic variation for smoking behaviors and nicotine dependence.. Clin Pharmacol Ther.

[41] Strasser AA, Malaiyandi V, Hoffmann E, Tyndale RF, Lerman C (2007). An association of CYP2A6 genotype and smoking topography.. Nicotine Tob Res.

[42] Li MD, Beuten J, Ma JZ, Payne TJ, Lou XY (2005). Ethnic- and gender-specific association of the nicotinic acetylcholine receptor alpha4 subunit gene (CHRNA4) with nicotine dependence.. Hum Mol Genet.

[43] Lou XY, Ma JZ, Payne TJ, Beuten J, Crew KM (2006). Gene-based analysis suggests association of the nicotinic acetylcholine receptor beta1 subunit (CHRNB1) and M1 muscarinic acetylcholine receptor (CHRM1) with vulnerability for nicotine dependence.. Hum Genet.

[44] Thorgeirsson TE, Geller F, Sulem P, Rafnar T, Wiste A (2008). A variant associated with nicotine dependence, lung cancer and peripheral arterial disease.. Nature.

[45] Berrettini W, Yuan X, Tozzi F, Song K, Francks C (2008). Alpha-5/alpha-3 nicotinic receptor subunit alleles increase risk for heavy smoking.. Mol Psychiatry.

[46] Amos CI, Wu X, Broderick P, Gorlov IP, Gu J (2008). Genome-wide association scan of tag SNPs identifies a susceptibility locus for lung cancer at 15q25.1.. Nat Genet.

[47] Hung RJ, McKay JD, Gaborieau V, Boffetta P, Hashibe M (2008). A susceptibility locus for lung cancer maps to nicotinic acetylcholine receptor subunit genes on 15q25.. Nature.

[48] Chanock SJ, Hunter DJ (2008). Genomics: when the smoke clears.. Nature.

[49] Weiss RB, Baker TB, Cannon DS, von Niederhausern A, Dunn DM (2008). A candidate gene approach identifies the CHRNA5-A3-B4 region as a risk factor for age-dependent nicotine addiction.. PLoS Genet.

[50] Saccone SF, Hinrichs AL, Saccone NL, Chase GA, Konvicka K (2007). Cholinergic nicotinic receptor genes implicated in a nicotine dependence association study targeting 348 candidate genes with 3713 SNPs.. Hum Mol Genet.

[51] Hunter DJ, Kraft P, Jacobs KB, Cox DG, Yeager M (2007). A genome-wide association study identifies alleles in FGFR2 associated with risk of sporadic postmenopausal breast cancer.. Nat Genet.

[52] Yeager M, Orr N, Hayes RB, Jacobs KB, Kraft P (2007). Genome-wide association study of prostate cancer identifies a second risk locus at 8q24.. Nat Genet.

[53] Pritchard JK, Rosenberg NA (1999). Use of unlinked genetic markers to detect population stratification in association studies.. Am J Hum Genet.

[54] Price AL, Patterson NJ, Plenge RM, Weinblatt ME, Shadick NA (2006). Principal components analysis corrects for stratification in genome-wide association studies.. Nat Genet.

[55] Prorok PC, Andriole GL, Bresalier RS, Buys SS, Chia D (2000). Design of the Prostate, Lung, Colorectal and Ovarian (PLCO) Cancer Screening Trial.. Control Clin Trials.

[56] Whitlock G, Lewington S, Mhurchu CN (2002). Coronary heart disease and body mass index: a systematic review of the evidence from larger prospective cohort studies.. Semin Vasc Med.

[57] Higgins JP, Thompson SG (2002). Quantifying heterogeneity in a meta-analysis.. Stat Med.

[58] Gauderman WJ, Morrison JL, Siegmund KD (2001). Should we consider gene x environment interaction in the hunt for quantitative trait loci?. Genet Epidemiol.

[59] Bierut LJ, Madden PA, Breslau N, Johnson EO, Hatsukami D (2007). Novel genes identified in a high-density genome wide association study for nicotine dependence.. Hum Mol Genet.

[60] Dudbridge F, Koeleman BP (2003). Rank truncated product of P-values, with application to genomewide association scans.. Genet Epidemiol.

[61] Wang K, Li M, Bucan M (2007). Pathway-Based Approaches for Analysis of Genomewide Association Studies.. Am J Hum Genet.

[62] Li M, Atmaca-Sonmez P, Othman M, Branham KE, Khanna R (2006). CFH haplotypes without the Y402H coding variant show strong association with susceptibility to age-related macular degeneration.. Nat Genet.

[63] Li MD, Cheng R, Ma JZ, Swan GE (2003). A meta-analysis of estimated genetic and environmental effects on smoking behavior in male and female adult twins.. Addiction.

[64] Kenfield SA, Stampfer MJ, Rosner BA, Colditz GA (2008). Smoking and smoking cessation in relation to mortality in women.. Jama.

[65] Lubin JH, Caporaso N, Hatsukami DK, Joseph AM, Hecht SS (2007). The association of a tobacco-specific biomarker and cigarette consumption and its dependence on host characteristics.. Cancer Epidemiol Biomarkers Prev.

[66] Munafo MR, Johnstone EC (2008). Genes and cigarette smoking.. Addiction.

[67] Fowler JS, Logan J, Wang GJ, Volkow ND (2003). Monoamine oxidase and cigarette smoking.. Neurotoxicology.

[68] Fowler JS, Logan J, Wang GJ, Volkow ND, Telang F (2005). Comparison of monoamine oxidase a in peripheral organs in nonsmokers and smokers.. J Nucl Med.

[69] Saccone NL, Rice JP, Rochberg N, Williams JT, Goate A (2002). Linkage for platelet monoamine oxidase (MAO) activity: results from a replication sample.. Alcohol Clin Exp Res.

[70] Wiesbeck GA, Wodarz N, Weijers HG, Dursteler-MacFarland KM, Wurst FM (2006). A functional polymorphism in the promoter region of the monoamine oxidase A gene is associated with the cigarette smoking quantity in alcohol-dependent heavy smokers.. Neuropsychobiology.

[71] Checkoway H, Franklin GM, Costa-Mallen P, Smith-Weller T, Dilley J (1998). A genetic polymorphism of MAO-B modifies the association of cigarette smoking and Parkinson's disease.. Neurology.

[72] Ragonese P, Salemi G, Morgante L, Aridon P, Epifanio A (2003). A case-control study on cigarette, alcohol, and coffee consumption preceding Parkinson's disease.. Neuroepidemiology.

[73] Tan EK, Chai A, Lum SY, Shen H, Tan C (2003). Monoamine oxidase B polymorphism, cigarette smoking and risk of Parkinson's disease: a study in an Asian population.. Am J Med Genet B Neuropsychiatr Genet.

[74] Ito H, Hamajima N, Matsuo K, Okuma K, Sato S (2003). Monoamine oxidase polymorphisms and smoking behaviour in Japanese.. Pharmacogenetics.

[75] Jin Y, Chen D, Hu Y, Guo S, Sun H (2006). Association between monoamine oxidase gene polymorphisms and smoking behaviour in Chinese males.. Int J Neuropsychopharmacol.

[76] Lewis A, Miller JH, Lea RA (2007). Monoamine oxidase and tobacco dependence.. Neurotoxicology.

[77] Tochigi M, Suzuki K, Kato C, Otowa T, Hibino H (2007). Association study of monoamine oxidase and catechol-O-methyltransferase genes with smoking behavior.. Pharmacogenet Genomics.

[78] Nilius B, Owsianik G, Voets T, Peters JA (2007). Transient receptor potential cation channels in disease.. Physiol Rev.

[79] Feng Z, Li W, Ward A, Piggott BJ, Larkspur ER (2006). A C. elegans model of nicotine-dependent behavior: regulation by TRP-family channels.. Cell.

[80] Gu Q, Lin RL, Hu HZ, Zhu MX, Lee LY (2005). 2-aminoethoxydiphenyl borate stimulates pulmonary C neurons via the activation of TRPV channels.. Am J Physiol Lung Cell Mol Physiol.

[81] Mwenifumbo JC, Tyndale RF (2007). Genetic variability in CYP2A6 and the pharmacokinetics of nicotine.. Pharmacogenomics.

[82] Nakajima M (2007). Smoking behavior and related cancers: the role of CYP2A6 polymorphisms.. Curr Opin Mol Ther.

[83] Haberl M, Anwald B, Klein K, Weil R, Fuss C (2005). Three haplotypes associated with CYP2A6 phenotypes in Caucasians.. Pharmacogenet Genomics.

[84] Tyndale RF, Sellers EM (2002). Genetic variation in CYP2A6-mediated nicotine metabolism alters smoking behavior.. Ther Drug Monit.

[85] Jin J, Cardozo T, Lovering RC, Elledge SJ, Pagano M (2004). Systematic analysis and nomenclature of mammalian F-box proteins.. Genes Dev.

[86] Rezvani K, Teng Y, Shim D, De Biasi M (2007). Nicotine regulates multiple synaptic proteins by inhibiting proteasomal activity.. J Neurosci.

[87] Caporaso NE (2007). Integrative study designs–next step in the evolution of molecular epidemiology?. Cancer Epidemiol Biomarkers Prev.

[88] Seminara D, Khoury MJ, O'Brien TR, Manolio T, Gwinn ML (2007). The emergence of networks in human genome epidemiology: challenges and opportunities.. Epidemiology.

[89] Rebbeck TR, Spitz M, Wu X (2004). Assessing the function of genetic variants in candidate gene association studies.. Nat Rev Genet.

