# Supplementary material for: Genome-Wide and Candidate Gene Association Study of Cigarette Smoking Behaviors
Source: PLoS One. 2009 Feb 27;4(2):e4653. doi: 10.1371/journal.pone.0004653 (PMC2644817; doi:10.1371/journal.pone.0004653)
Supplement: Table S2 — (0.03 MB DOC) [file pone.0004653.s002.doc]

Supplementary Table 2. Pearson r between continuous smoking behaviors within NHS (above diagonal) and PLCO (below diagonal). All P<0.0001 except as noted.

|  | **CPD** | **SMKDU** | **SMKAGE** | **PKYRS** |
| --- | --- | --- | --- | --- |
| **CPD** | * | 0.16 | -0.05^†^ | 0.70 |
| **SMKDU** | 0.22 | * | -0.15 | 0.82 |
| **SMKAGE** | -0.18 | -0.28 | * | -0.13 |
| **PKYRS** | 0.78 | 0.78 | -0.31 | * |

^†^P=0.11

CPD= cigarettes per day

SMKDU= duration of smoking

SMKAGE= age at smoking initiations

PKYRS= pack-years
