# Supplementary material for: Genome-Wide and Candidate Gene Association Study of Cigarette Smoking Behaviors
Source: PLoS One. 2009 Feb 27;4(2):e4653. doi: 10.1371/journal.pone.0004653 (PMC2644817; doi:10.1371/journal.pone.0004653)
Supplement: Table S3 — (0.05 MB DOC) [file pone.0004653.s003.doc]

Supplementary Table 3. Meta-analysis of three SNPs tested for association to CPD.

| **Dataset** | | **N smokers** | **P-value for association with CPD** | | |
| --- | --- | --- | --- | --- | --- |
|  | |  | **rs1051730** | **rs8034191** | **rs16969968** |
| NHS | | 1244 | 1×10^-3^ | 1×10^-3^ | 1×10^-3^ |
| PLCO | | 1373 | 0.10 | 0.06 | 0.06 |
| Hung (ref. 47) | | 847 |  |  | 0.485 |
| Thorgeirsson (ref. 44) | Iceland | 13,945 | 1x10^-18^ | 1×10^-15^ |  |
|  | Spain | 523 | 0.32 |  |  |
|  | Netherlands | 1,375 | 0.02 |  |  |
| Amos (ref. 46) discovery: former smokers | Controls | 657 | 0.02 | 0.003 |  |
|  | Lung cancer Cases | 603 | 0.05 | 0.053 |  |
| Amos discovery: current smokers | Controls | 510 | 0.40 | 0.476 |  |
|  | Cases | 551 | 0.20 | 0.164 |  |
| Amos replication (TX): former smokers | Controls | 283 | 0.02 | 0.024 |  |
|  | Cases | 399 | 0.14 | 0.056 |  |
| Amos replication (TX): current smokers | Controls | 349 | 0.09 | 0.177 |  |
|  | Cases | 312 | 0.67 | 0.708 |  |
| Amos replication (UK): former smokers | Controls | 1228 | 0.40 | 0.282 |  |
|  | Cases | 1324 | 0.03 | 0.032 |  |
| Amos replication (UK): current smokers | Controls | 704 | 0.08 | 0.051 |  |
|  | Cases | 562 | 0.22 | 0.775 |  |
| Combined p-value^1^ | | | 5×10^-32^ | 2×10^-29^ | 2×10^-3^ |
| Combined p-value, excluding Thorgiersson | | | 5×10^-9^ | 3×10^-7^ | 2×10^-3^ |

^1^Using Stouffer's weighted z-score method (ref. 56).
