# Supplementary figures and images for: Genome-Wide and Candidate Gene Association Study of Cigarette Smoking Behaviors
Source: PLoS One. 2009 Feb 27;4(2):e4653. doi: 10.1371/journal.pone.0004653 (PMC2644817; doi:10.1371/journal.pone.0004653)

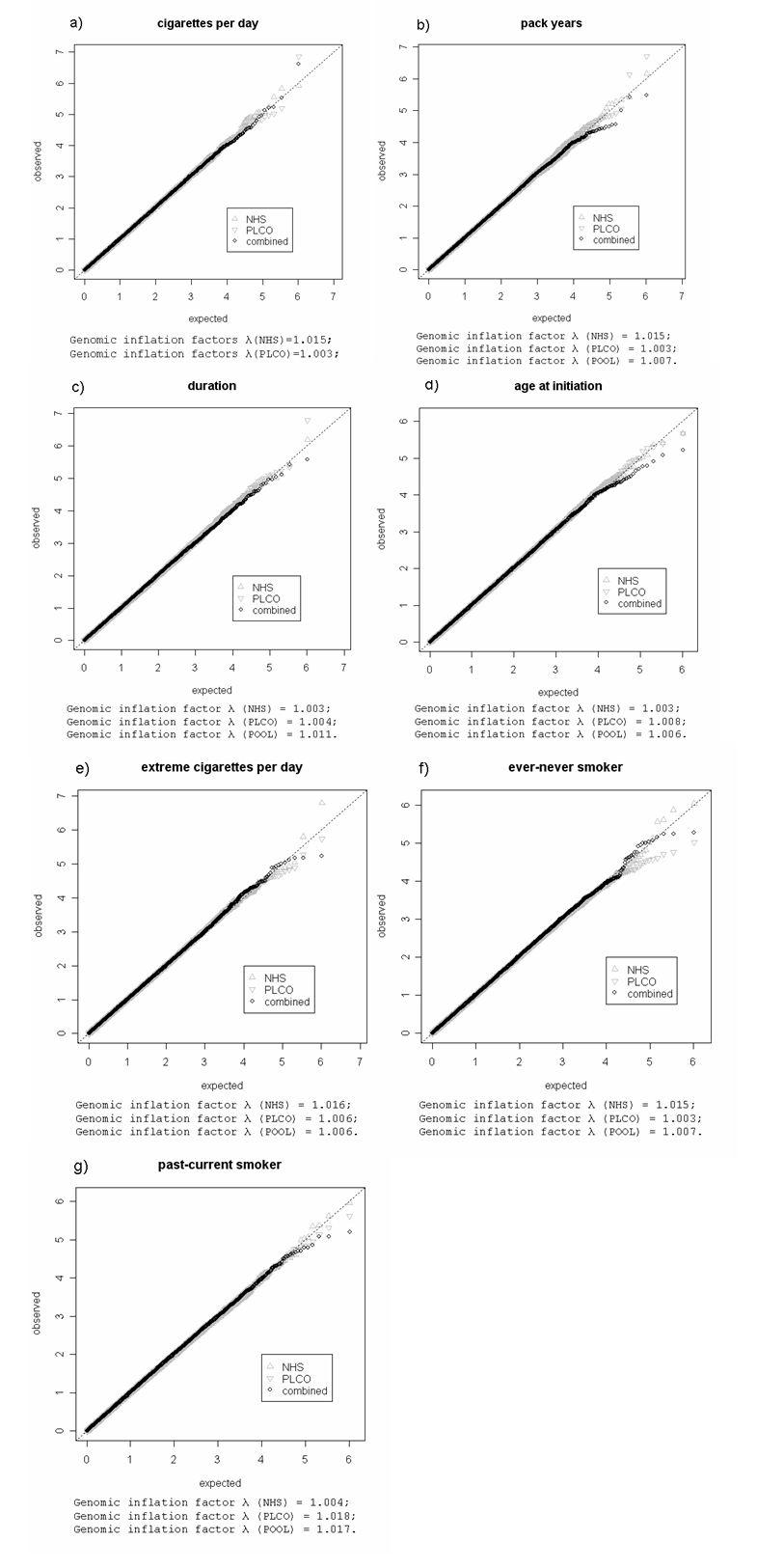

Supplement: Figure S1 — (0.43 MB TIF) [file pone.0004653.s004.tif]

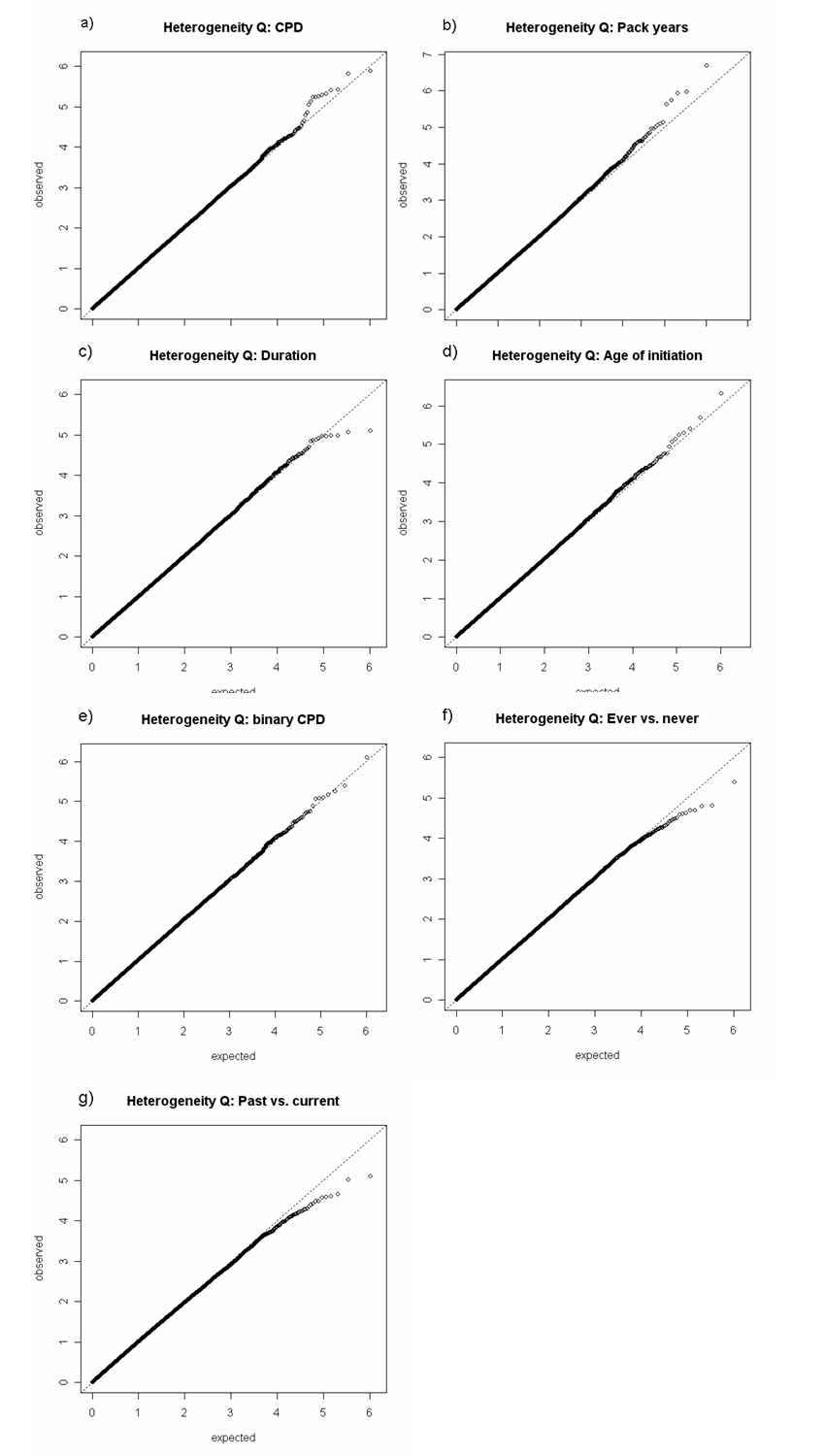

Supplement: Figure S2 — (0.31 MB TIF) [file pone.0004653.s005.tif]
